# Orientation control of ideal blue phase photonic crystals

**DOI:** 10.1038/s41598-020-67083-6

**Published:** 2020-06-23

**Authors:** Eva Otón, Hiroyuki Yoshida, Przemysław Morawiak, Olga Strzeżysz, Przemysław Kula, Masanori Ozaki, Wiktor Piecek

**Affiliations:** 1 Institute of Applied Physics, Military University of Technology, gen. S. Kaliskiego 2, 00-908 Warsaw, Poland; 2 Division of Electrical, Electronic, and Information Engineering, Osaka University 2-1 Yamadaoka, Suita, Osaka, 565-0871 Japan

**Keywords:** Photonic crystals, Liquid crystals, Self-assembly

## Abstract

Three-dimensional (3D) photonic crystals like Blue Phases, self-assemble in highly organized structures with a sub-micrometer range periodicity, producing selective Bragg reflections in narrow bands. Current fabrication techniques are emerging at a fast pace, however, manufacturing large 3D monocrystals still remains a challenge, and controlling the crystal orientation of large crystals has not yet been achieved. In this work, we prepared ideal 3D Blue Phase macrocrystals with a controlled crystal orientation. We designed a method to obtain large monocrystals at a desired orientation and lattice size (or reflection wavelength) by adjusting the precursor materials formulation and a simple surface treatment. Moreover, using the same method, it is possible to predict unknown lattice orientations of Blue Phases without resorting to Kossel analysis. Producing large 3D photonic crystals that are also functional tunable structures is likely to have a direct impact on new photonic applications, like microcavity lasers, displays, 3D lasers, or biosensors.

## Introduction

The blue phases (BP) of cholesteric liquid crystals have become an excellent approach for novel applications in photonic devices because of their very particular properties and structure^1–3^. BPs exhibit a highly organized 3D structure with a sub-micrometer range periodicity, as opposed to other liquid crystal phases. This 3D organization is achieved by the self-assembly of the liquid crystal molecules into periodic cubic structures that produce bright selective Bragg reflection in narrow bands and are optically isotropic. However, producing ideal BP crystals in large volumes remains a challenge. BPs have been previously produced as monocrystals by various methods, for instance, by orienting layers^4^, by long lasting applied voltage^5^, microwell confinement^6^, temperature gradient^7^ or photopatterning^8^.

Despite the great progress in techniques and materials, producing ideal BP crystals in large volumes is still problematic, since the current produced BP crystals are oftentimes polycrystalline (platelet structure), the single crystal size is limited (in the micrometer range) or the BP liquid crystal is not stabilized in temperature, among other issues. In addition, controlling the growth of large BP crystals with a particular lattice plane orientation has just been started to be explored, for instance by using patterned substrates to assist the nucleation and growth of BP crystals in small areas^8–10^.

In this work, we obtained ideal BP macrocrystals in large volumes. We show that it is possible to tailor the resulting lattice orientation by changing the mixtures formulation, using the a simple surface treatment that induces weak anchoring energy. We propose a method to obtain BP ideal macrocrystals in a desired BP lattice orientation and reflection wavelength. Finally we prepared several ideal BP crystals at specific orientations by this method and stabilized them at room temperature.

In addition, the same method can be employed for predicting unknown BP crystal lattice orientations without the need of Kossel analysis.

A BP macrocrystal can be considered as a 3D photonic crystal, since it shows a periodic cubic structure with a bandgap in the visible wavelength range. For assessing the homogeneity and orientation of the macrocrystal, optical methods and analysis with Kossel diagram simulations were employed.

## Results

**Figure 1:**
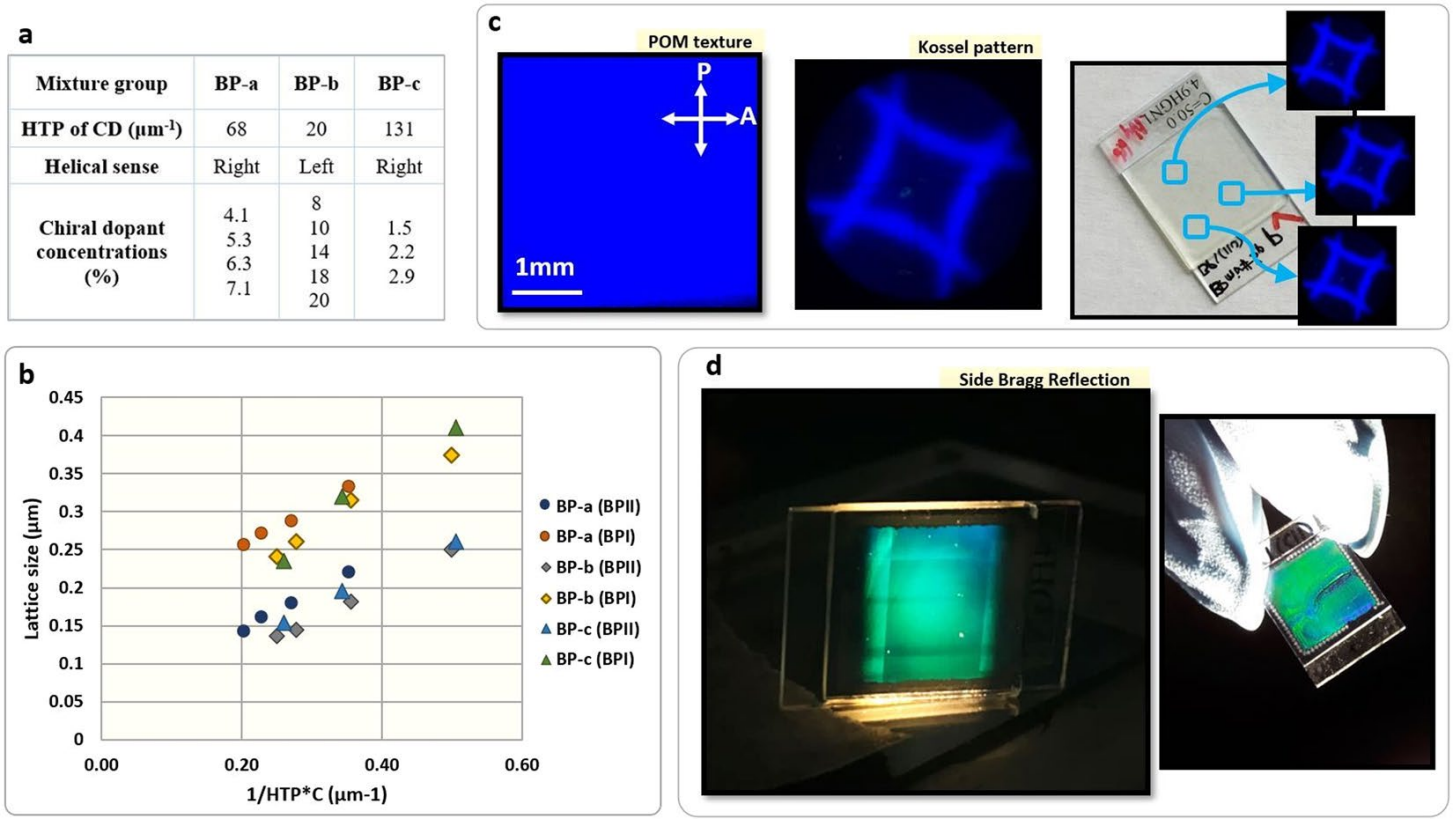
Blue Phase (BP) mixtures and BP monocrystal preparation. (**a**) Composition of the three groups of BP mixtures. (**b**) BP lattice size relationship with chiral dopant (CD) concentration and HTP. (**c**) BP crystal texture (field of view: ≈14 mm^2^), its corresponding Kossel pattern and stabilized BP in a glass cell, as seen by POM in reflection mode and crossed polarizers. (**d**) Bragg reflection produced by the BP monocrystal when the cell is side-illuminated (in the cells, BP crystal is 1 cm^2^).

Three groups of cholesteric liquid crystal mixtures (named *BP-a*, *BP-b* and *BP-c* for simplicity) that display BPs in certain temperature ranges were prepared. Each group is composed by a host nematic liquid crystal mixture base, a chiral dopant (CD) and a mesogenic monomer mixture. The same host nematic mixture was used for all groups so that only the CD type was different among groups (see Methods). Within each group, a number of sub-mixtures were prepared possessing different concentrations of the CD. Figure 1a summarizes the mixtures compositions used in this study.

Each mixture was introduced into a sandwich cell assembled from glass substrates coated with rubbed polyamides (Nylon 6). The cell-gap was set to either 5 or 15 µm and the BP orientations were characterized by polarized optical microscopy (POM), Kossel pattern analyses, and reflection spectroscopy. Between crossed polarizers in reflection microscopy, vibrant colors are observed corresponding to the Bragg reflection wavelengths of the BP. For a BP with lattice constant of *a*, and refractive index of *n*, the Bragg reflection wavelength for a crystal plane with Miller index (*hkl*), λ_hkl_ is given by ${\lambda }_{hkl}=\frac{2na}{\sqrt{{h}^{2}+{k}^{2}+{l}^{2}}}$; since *a* is typically a few 100 nm and n ~ 1.6, λ_hkl_ appears in the visible to the ultraviolet part of the spectrum. Moreover, Kossel patterns, which are diffraction patterns obtained for convergent monochromatic light rays, provide information about the specific orientation of the BP cubic structure, its phase (BPI or BPII) and the order parameter in the BP crystal^11^. The two techniques combined enable the characterization of the crystal size and its orientation with respect to the glass substrates.

The relationship between the lattice size and the helical twisting power (HTP) and concentration product (HTP*C) of the CD was evaluated by analyzing the reflection wavelengths observed for all mixtures. The inversely proportional relationship between the pitch and the HTP*C is well known in cholesteric liquid crystals; however, this relationship had not been confirmed in BPs, where a discontinuous jump in pitch length is known to occur between phases. Figure 1b shows the lattice size of the BP observed in experiment plotted against the product of the HTP and CD concentration. Each color corresponds to one group of BP mixtures (BP-a, b or c) and either the BPI or the BPII observed for all the CD concentrations. A correlation between the lattice size and HTP*C appears. Certainly, as expected, higher CD concentrations produce smaller lattice constants, but what is more significant is that the trend seems to be independent of the CD type, and all BPIs and all BPIIs tend to be grouped together - a factor difference between both. The calculated trend fit follows the following relationship: lattice size, a = m/HTP*C + ***n***, where *m =* 0.5 ± 0.05 for both BPII and BPI, and *n* = 0.03 ± 0.02 for BPII and *n* = 0.15 ± 0.02 for BPI.

As a result, it would be possible to calculate the value of *h*^*2*^ + *k*^*2*^ + *l*^*2*^ in any given observed BP phase without resorting to Kossel pattern analysis. For example: one of our BP phases produced a reflection peak at 644 nm, and the original mixture was prepared with a 5.3% of CD whose HTP was 68 µm^−1^. Applying the analytical trend, the calculated lattice constant is *a* = 288 nm. Therefore, by: $\lambda =\frac{2na}{\sqrt{{h}^{2}+{k}^{2}+{l}^{2}}}$, h^2^ + k^2^ + l^2^ = 2.039 ≈ 2, and (*hkl*) = (110), which was corroborated by its Kossel pattern, as well. The calculated *hkl* fitted with the experimentally ones for all the observed phases.

Interestingly, we found that there is a correlation between the lattice plane orientation of BPs and CD concentration. In particular, single ideal crystals of BP were obtained in mixtures with the lowest CD concentration for each CD type. Cooling from isotropic phase, the grown BP crystal shows homogeneity along the whole cell and a single distinctive reflection color, thus suggesting that the whole BP cubic structure is organized and oriented in a unique 3D crystal. Figure 1c shows the POM texture in reflection between crossed polarizers of a stabilized BP in a glass cell and its Kossel pattern, corresponding to the phase BPI with the (200) plane orientated parallel to the substrates. Hereafter, all BP phases are observed in reflection mode, with the same POM configuration of polarizer and analyzer, and the photographs of BP textures were observed with the same field of view (≈ 14 mm^2^ except were indicated). Also, the notation BP_(*hkl*)_ is used to refer to BP crystals with (*hkl*) lattice plane orientation. The observed Kossel pattern in the Figure is clear and sharp, and does not change, rotate, or become dimmer upon changing the observation spot in the glass cell. The resulting Kossel pattern is an average of the orientation changes and local variations in the (*hkl*) lattice planes^11,12^, thus suggesting that there is a single BPI crystal with a unique lattice orientation and a fixed azimuthal angle in our glass samples, since Kossel diagrams become blurry upon introduction of crystal misorientations. The stabilized BP crystal is highly transparent, as shown in cell at the right image in Fig. 1c, with a transmission T = 85%, (measured outside the photonic bandgap, @550 nm). The BP crystal was polymer stabilized, remaining as BP in a temperature range over 70 K.

The crystal uniformity can be also assessed by checking side illumination Bragg reflection. Unlike cholesteric liquid crystals, which only present Bragg reflection in one dimension, BPs, being periodic in three dimensions, can produce multiple reflections in different directions^13,14^. When we illuminate our BP crystal from the side of the glass cell using a white light source, a bright side Bragg reflection is produced, reflecting light through the glasses of the cell. Upon increasing the observation angle with respect to the illumination source direction, the reflected wavelengths become shorter, following the Bragg condition. Figure 1d (left) shows the side Bragg reflection from single crystal stabilized BP cells: note that there is a single distinctive reflection color with the same intensity along the cell. While the crystal is side-illuminated, it is possible to study its homogeneity because if part of the BP is positioned in a slightly different lattice orientation, it will reflect the light differently and a defect will be spotted. Figure 1d (right) shows a stabilized BP cell with a defect in its center, where the BP cubic lattices are oriented in a different position. These defects are corroborated by a rotated or blurry Kossel pattern, as well.

**Figure 2:**
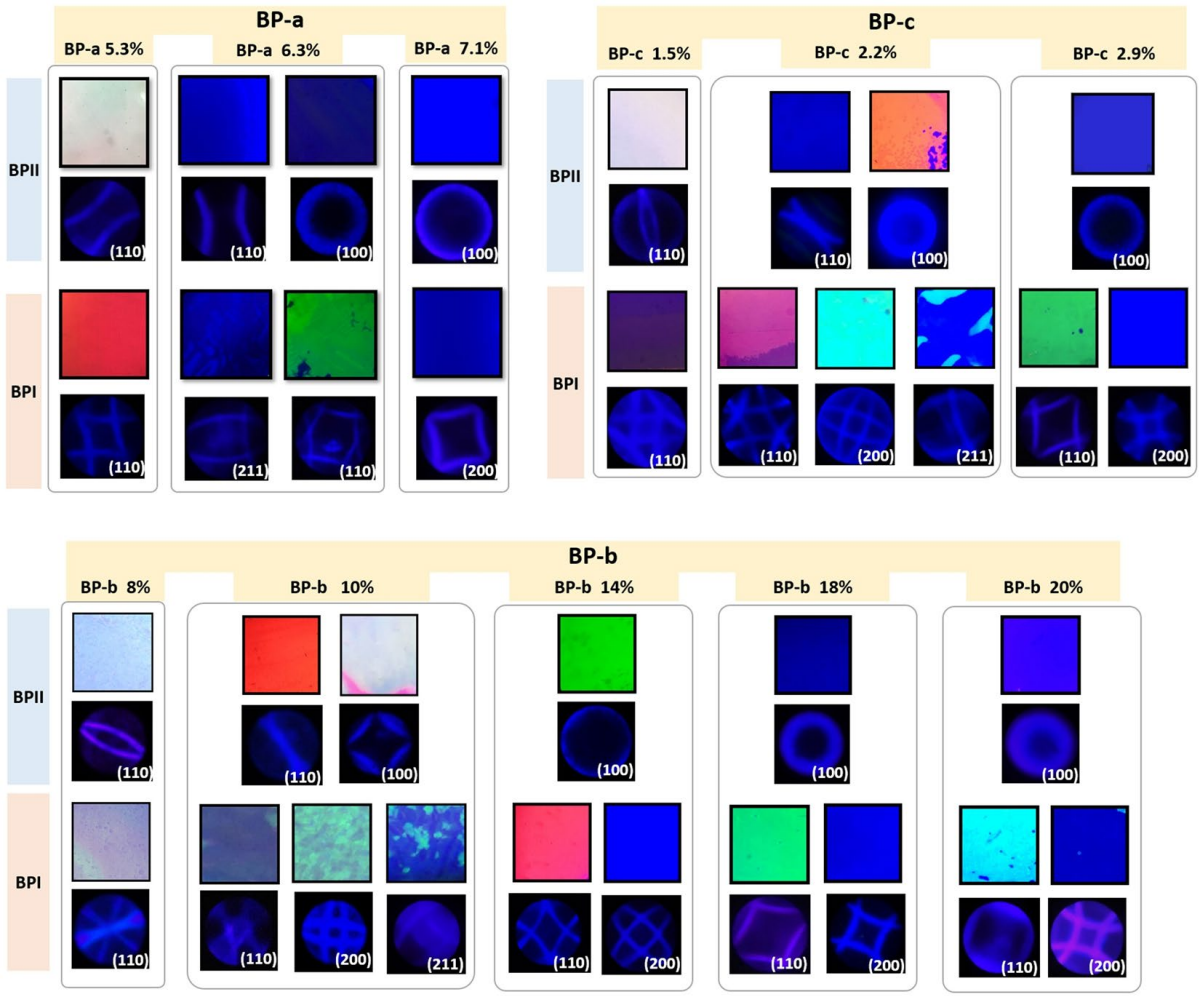
POM images and corresponding Kossel patterns for each BP phase observed in the three mixture groups. BP phases and lattice orientations displayed by each BP group was observed with different CD concentrations in 5 µm cells. With increasing the CD concentrations one can observe progressively smaller lattice sizes for the same lattice orientation (reflection color shifting to blue) and larger Kossel patterns, corresponding to larger diffraction angles. (Field of view: 14 mm^2^, except for BPI’s phases of BP-b 10%, and BP-c 2.2% ~2 mm^2^).

Figure 2 shows the POM images for all BPI and BPII phases displayed by the three groups of mixtures and corresponding Kossel patterns. The preferential lattice plane orientation on the substrate changed when the CD concentration was varied. With each increasing step of CD concentration, different BP lattice orientations seemed to have predominance over others. For the highest and lowest CD amounts in mixture group *BP-a*, a single lattice orientation has a clear dominance. For mixture *BP-a 5*.*3%*, both BPII and BPI lattices are oriented with the [110] direction. The mixture with the highest CD concentration, 7.1 wt%, produced monodomain BPII (100) and BPI (200). It is worth mentioning that, in both cases, the favored lattice orientation covered the whole extent of the glass cell, displaying a unique Kossel pattern with a constant azimuthal angle.

**Figure 3:**
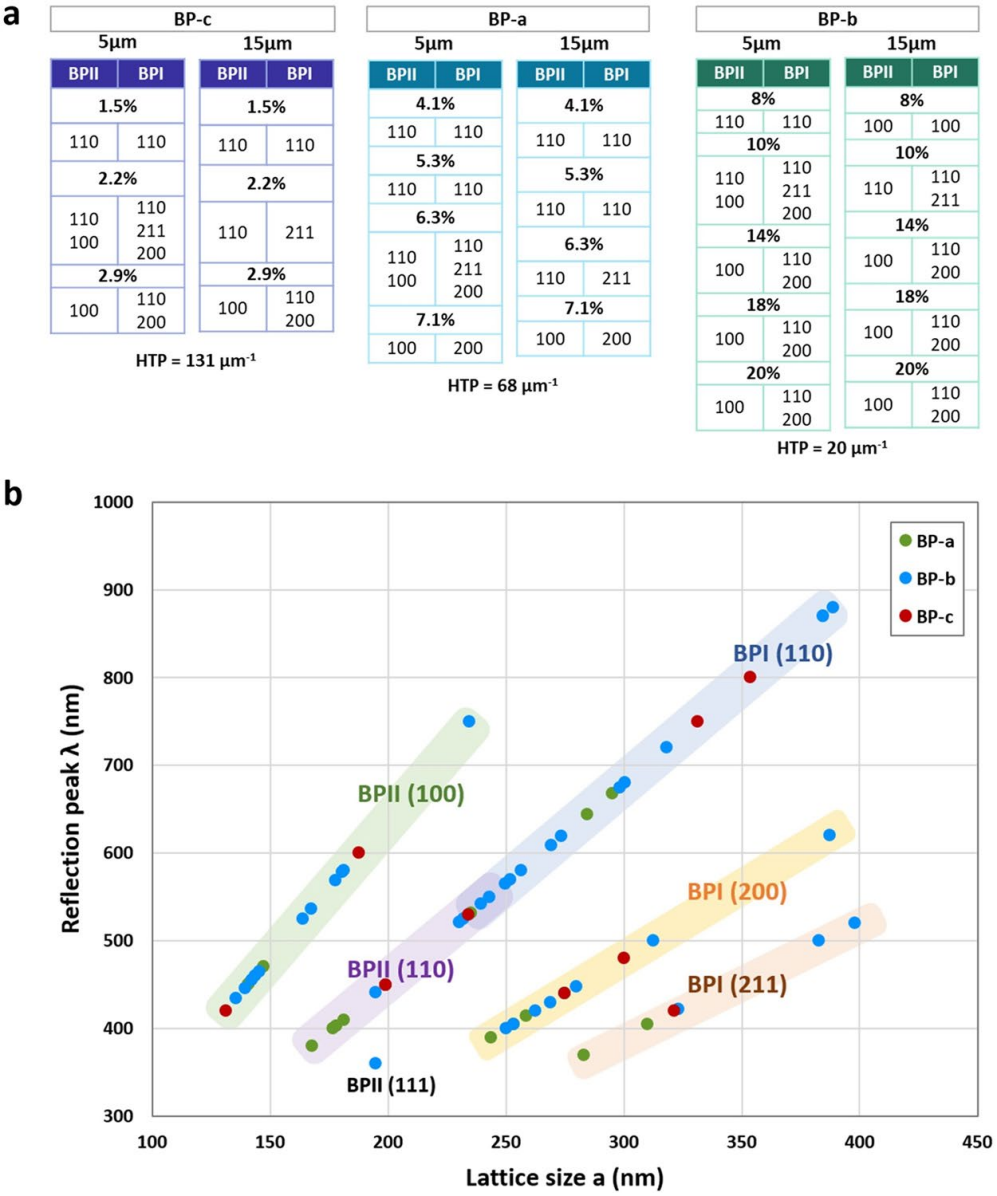
Relationship between BP crystal orientation and reflection wavelength. (**a**) Summary of all the BP lattice orientations observed in the three groups of mixtures, depending on the CD concentration, and arranged by the CD HTP for 5 and 15 µm thick cells. (**b**) Relationship between the Bragg reflection wavelength measured for every crystal lattice orientation and the lattice size for all observed BP phases. Each crystal lattice orientation is allowed within a reflection wavelength-lattice size range following a linear trend.

The same orientation behaviors were observed for mixtures *BP-b* and *BP-c*. For low CD concentrations, (110) was the only induced orientation for both BPII and BPI, and even while steadily decreasing the CD amount until no BP was formed at all, (110) orientation prevailed. Figure 3a summarizes the lattice plane orientation observed for the three types of mixtures with different CD concentrations in 5 and 15 µm cells. As the table shows, the lowest CD concentrations induce monodomain orientation of BPII (110) and BPI (110). At intermediate CD concentrations, (*BP-a* 6.3%, *BP-b* 10% and *BP-c* 2.2%), there is a coexistence of several lattice orientations that cover large areas of the cell: BPII (100), BPI (211) and BPI (200). Some coexisting phases disappear at larger thicknesses, where mostly BPII (110) and BPI (211) appear. Then, when reaching higher CD concentrations BPII (100) and BPI (200) become dominant, although *BP-b* and *BP-c* showed the appearance of BPI (110), (*BP-b* 18 and 20%, showed considerably less amount of BPI (110) compared with the samples with less CD concentration).

For every BP phase observed, the corresponding Kossel pattern showed a fixed azimuthal angle that did not rotate or change in other cell spots, thus demonstrating monocrystalline BP phases. The only exception was phase BPI (211), which showed slight changes in the azimuthal angle and dimmer Kossel patterns. The observed Kossel patterns and corresponding lattice sizes were corroborated by our Kossel pattern simulator (see SI).

### Tailoring the BP lattice orientation and reflection band

Upon varying the CD composition and the cell gap similar lattice orientations and lattice constants were observed in all mixtures. However, not all lattice orientations appear at any reflection wavelength or lattice size. By understanding where the lattice orientations are allowed, we could design a particular BP ideal crystal at any orientation. First, choosing a BP orientation in a desired reflection wavelength, and then, with the resulting lattice size and by the trend showed in Fig. 1b, we could calculate what is the HTP and CD concentration product needed to prepare the required liquid crystal mixture.

Figure 3b shows a map of the BP lattice orientations: the reflection wavelength vs. the lattice size for every lattice orientation that was observed in all three groups for both 5 and 15 µm cells. We measured the Bragg reflection wavelength for each BP lattice orientation, and then calculated their corresponding lattice size, *a*, corroborated by the Kossel pattern simulations. Each data point color corresponds to one BP mixture group (BP *a*, *b* or *c*). A map of lattice orientations becomes clear, upon adding more phases, independently of the CD type that was used. The same lattice orientations are grouped together in the same area of the graph on a linear trend, the slope being *2n/h*^*2*^ + *k*^*2*^ + *l*^*2*^, following $\lambda =\frac{2na}{\sqrt{{h}^{2}+{k}^{2}+{l}^{2}}}$. For instance, all BPI (110) group together with relatively high reflection wavelengths and lattice sizes, while at low reflection wavelengths only BPII (110) or BPI (211) orientations appear. This is in concordance with the observed samples, as low CD concentrations tend to produce red reflections while high concentrations produce blue reflections. BPII (111) was observed only in one mixture group, and in very limited amounts. In addition, around lattice size *a* = 240 nm, both BPII (110) and BPI (110) are allowed, although those BP phases fall at the very extremes of CD concentrations, (BPI by excess and BPII by defect), since a CD with a low HTP can produce BPII with a large lattice constants and vice versa.

Naturally, by using this method in the opposite way, the lattice orientation of any given BP crystal can be predicted without using Kossel diagrams: given a BP phase and measuring the reflection peak, the lattice size is calculated as shown above. Then, using the *hkl map*, the lattice orientation that is allowed for that particular *λ* and *a* combination can be determined.

**Figure 4:**
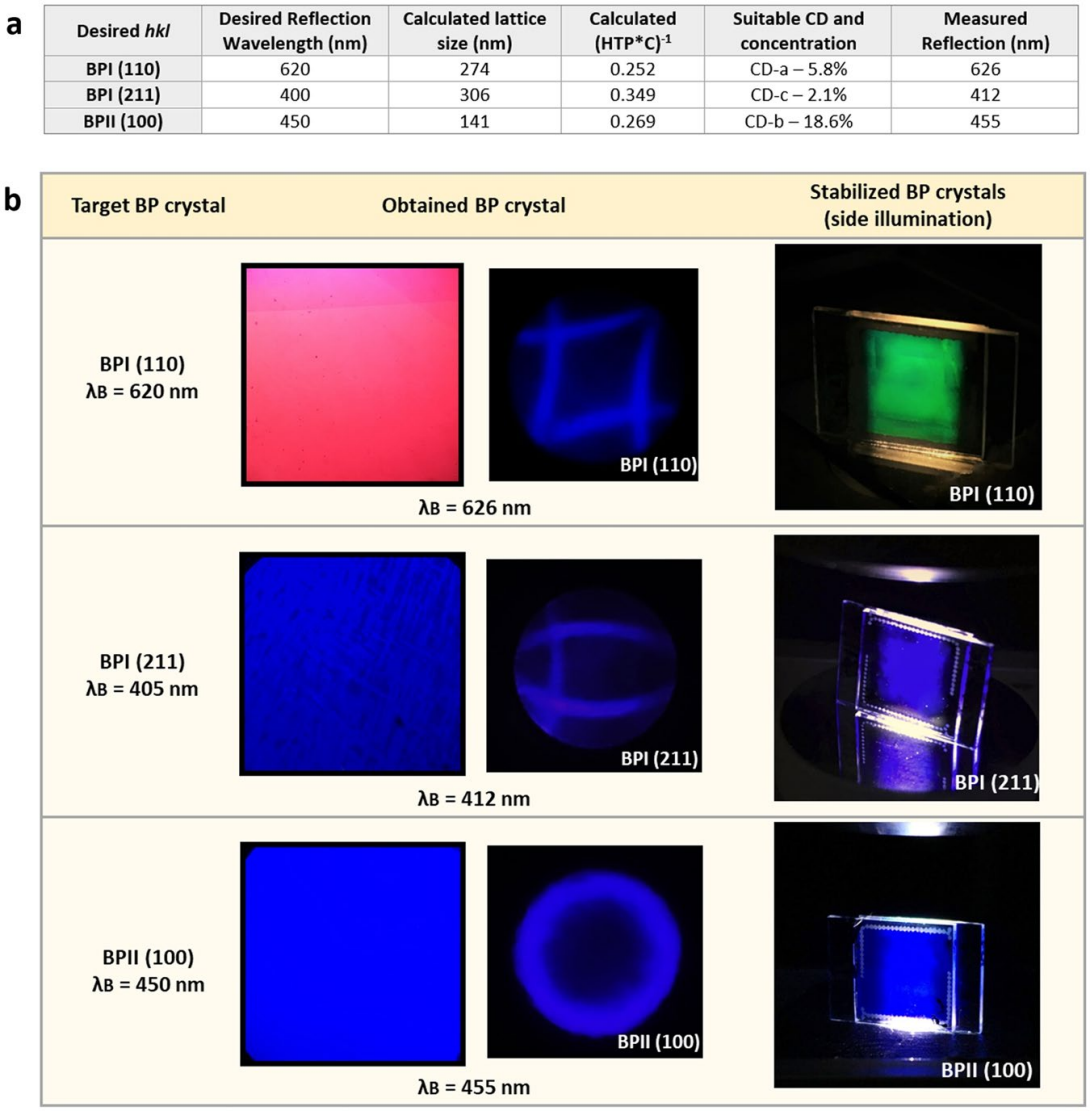
Design and control of the resulting BP crystal orientation. (**a**) Calculation of the chiral dopant concentration needed for designing the proposed BP ideal crystals. (**b**) Stabilized BP monocrystals obtained at a desired lattice orientation and reflection wavelength. The micrographs show the POM images and corresponding Kossel patterns achieved for each designed crystal orientation and reflection wavelength. When side-illuminated, the BP crystals show a uniform structure with a bright single colored Bragg reflection. POM field of view is 14 mm^2^, cell active area is 1 cm^2^, and all BP crystals have been stabilized at room temperature.

We chose to design and prepare 3 different BP monocrystals. The *hkl map* shows the allowed areas where particular lattice orientations and reflection wavelengths appear, thus, within the allowed range, we chose 3 different lattice orientations and reflection wavelengths. The calculation and selection of the most suitable CDs to produce those BP crystals is summarized in Fig. 4a. Certainly, it is desirable to avoid CD concentrations where a coexistence of different lattice orientations is possible, so the CD type and cell gap have been selected accordingly.

Figure 4b shows the BP monocrystals that were obtained by the proposed designing method. All BPs have been polymer stabilized and are stable at room temperature. Cell gap was set to 5 µm for BPI (110) and BPII (100) and to 15 µm for BPI (211). BP textures and Kossel patterns were measured after stabilization, as well as the final reflection wavelengths (Fig. 4a).

It is worth mentioning that matching the obtained reflection wavelength with the proposed one is also affected by other factors other than the selected CD and its concentration. The obtained BP crystal lattice sizes were slightly larger than the prediction, because the exact temperature where the BP is stabilized plays a role in the final reflection wavelength, as well. BP lattice sizes vary within the temperature ranges where they appear: BPII decreasing and BPI increasing as the temperature decreases. Polymerization conditions, like UV exposure intensity, wavelength, or stabilization temperature can affect the resulting polymer network. As a result, the polymerization process itself has an influence as the polymer network forms through the BP disclination lines, fixing the structure, causing slight variations in the final lattice size^15^.

Note that the side illuminated glass cells produce different single colored bright reflections. The reflection wavelengths follow the Bragg rule and depend on the specific position of the crystalline planes and angle of observation. By checking the side Bragg reflection on the glass cells, the homogeneity of the layers is revealed. In case of BPI (211), the layer showed some inhomogeneities, and the cell edges were stabilized in cholesteric phase, and thus the layer edges do not show any side Bragg reflection (Fig. 4b, BPI (211) right). Both BPI (110) and BPII (100) were obtained as virtually perfect crystals, showing an excellent homogeneity of the layer with a bright reflection and without noticeable defects in their layers or any changes in Kossel pattern intensity.

## Discussion

In this work, we prepared ideal Blue Phase macrocrystals and tailored their lattice orientation by modifying the BP precursor mixture formulation and by using weak anchoring conditions on the layer surfaces. We propose a simple method to obtain ideal Blue Phase crystals at a desired orientation and reflection wavelength. Our process demonstrates considerable progress in the manufacturing of 3D photonic crystals, which is a particularly demanding process, as there are numerous constraints for achieving large and homogeneous crystals^16^. Previous works show other innovative techniques and materials to manufacture photonic crystals, such as multi-exposure two-beam interference technique^17^, or direct laser write lithography in photoresists^18^.

The dependence of the orientation on the CD concentration is a surprising result, since previous research showed that the control over the lattice orientation can be achieved in small areas only by entirely changing the glass cell surface parameters^19^. However, in our case, all glass cells underwent the same manufacturing protocol, with a simple rubbing surface treatment.

The change in CD clearly induced a change in the preferred lattice orientation displayed by the BP. For each CD concentration, the interaction between BP and substrate changes, so the surface would only be able to induce one particular orientation. The anchoring energy would be the driving force to produce the change in lattice orientation. The intrinsic mechanism is consistent with previous research^9^, where the interfacial free energy dominates certain lattice orientations while limiting others as the anchoring energy varies at the interface.

For the surface treatment, instead of employing conventional polyimide alignment layers, generally used for aligning liquid crystals, we used polyamide layers: Nylon 6 and Nylon 6-6. Polyamides have been used almost exclusively for smectic liquid crystals – ferro- and antiferroelectric liquid crystals (FLC, AFLCs) – as these alignment layers possess weak anchoring energies and produce excellent alignment and electrooptical behavior^20,21^. On the other hand, conventional polyimides fall into the category of strong anchoring layers, with anchoring energies, *W*, in the order of *W* ≈ 10^−4^ J/m^2^, while for weak anchoring layers, like Nylon and photoalignment layers, or soft-rubbed layers, anchoring energies can reach one or two orders of magnitude lower^22,23^. The weak anchoring conditions of our Nylon layers could be an important factor in producing a uniform monocrystal with a unique lattice orientation and azimuthal angle. We note that the use of other alignment layers did not induce monodomain alignment, as we show in the Supplementary Information (SI).

Given that our work is focused on manufacturing high quality 3D photonic BP crystals, it is worth considering the introduction of defects into the crystalline structure. Small crystal misorientations or defects are not observable by the conventional methods employed for studying BPs. Kossel diagram analysis or POM are insufficient to detect small crystalline defects. Side Bragg reflection, on the other hand, was, surprisingly, an excellent method to detect disorganized lattice areas in the BP crystals. Small defects are shown amplified by the light reflected through the BP crystals. Unorganized crystals produce scattering when observed by side illumination and can be seen by the naked eye as bright spots or different colored areas. However, local lattice defects in the crystalline structure will be too small to be detectable by optical methods, thus requiring a deeper look into the crystal bulk. As a result, we are currently performing research on this topic, especially focusing on larger samples.

Phase transitions between all BPII and BPI lattice orientations were analyzed as well. BPI phases were always obtained from a BPII, as the phases were checked while cooling down the mixtures. A given BPI lattice orientation was always obtained by one of the two following paths starting at a particular BPII. A BPII in lattice orientation BPII (100) became either BPI (200) or BPI (110). For BPII (110), the obtained BPI was either BPI (110) or BPI (211). Every phase transition observed in all three mixture groups showed either of the two paths. These results suggest that there is a relationship between particular BPII and BPI lattice orientations. The reason behind these specific phase transitions could be related to the close proximity of the symmetry between particular lattice orientations and a correspondence between distinct crystal axes. For instance, BPII (100) is closer by symmetry to BPI (200), than to BPI (211), the energy barrier required for rearranging the BPII (100) structure would be higher compared to rotating to (211). Moreover, transitions like BPII (100) → BPI (211), and BPII (110) → BPI (200) were never observed in any of the three mixture groups. Nevertheless, in order to confirm these hypotheses, further theoretical research with actual crystallographic data would be required. Of course, it should also be taken into consideration the structural phase change between simple cubic (SC) and body centered cubic (BCC) corresponding to the transition between BPII and BPI structures respectively. This theoretical analysis is out of the scope of the present paper, however, further research is ongoing on this topic at the moment.

By using the map of *hkl* lattice orientations and relationship with the chiral dopant it is possible to determine what is the chiral dopant concentration and HTP required to achieve a particular lattice orientation. The selection of the most suitable CD for each BP is essential in order to avoid the appearance of more than one crystal orientation. In addition, using the same tools it is possible to predict an unknown lattice orientation of a given BP phase without the need of using a Kossel pattern analysis set-up.

## Methods

### Mixtures

All three groups of BP mixtures contained a nematic host mixture and different chiral dopants on each group. The host molecules were synthesized in our facilities at WAT, whose major compositions are fluorinated terphenyls, biphenyl, cyclohexylbiphenyls, bicyclohexylbiphenyls and difluoromethyl-oxy-phenyls. The nematic host mixture was doped with a chiral dopant, *a*, *b* or *c*, where: *a* = ISO(6OBA)_2_ (Midori Kagaku Co. Ltd.), *b* = [1,1;4,1] terphenyl-4,4-dicarboxylic acid bis-(1-methylheptyl) ester (synthetized at our facilities^24^), *c* = R5011 (Merck, KGaA). All three groups of mixtures were prepared with a monomeric mixture (9.0%) consisting of RMM34C (Merck KGaA) and an in-house synthesized monofunctional monomer compound at a (1:1) ratio.

### Ideal BP crystal preparation

Glass cells consisted of two ITO coated ultraflat glass substrates assembled into a sandwich cell of 5 or 15 µm thick. The ITO coated glass substrates were prepared with Nylon 6 polyamide, thermally conditioned, and rubbed in antiparallel configuration. Then, BP mixtures were filled into the cells by capillarity in isotropic phase. Polyamide Nylon 6, as well as Nylon 6-6, produced perfect ideal and oriented BP crystals. As a comparison, in addition to Nylon layers, a number of polyimide layers with known strong anchoring conditions were tested as alignment for our BP mixtures. None of these alignment layers produced monocrystalline BP oriented layers though; the strong anchoring present in most polyimide alignment layers produced platelet BP layers and disorganized lattice orientations in all cases (see comparison in SI).

All BP phases underwent a full thermal cycle and then analyzed while cooling down from isotropic state to cholesteric liquid crystal in an Instec HCS402 hot stage platform and STC 20U thermal controller. BP phases were analyzed by POM in reflection mode in an Olympus BX51 with a 5×/0.15 objective. Kossel patterns were obtained in conoscopic configuration of the microscope using a 60×/0.70 objective with a 450 nm light source. BP ideal crystals were polymer stabilized with a 365 nm (Hamamatsu Lightningcure LC8) light source for 35 minutes at 0.4 W/cm^2^. BP reflection spectra were measured with Ocean Optics Flame-T spectrometer.

### Kossel pattern simulations

Kossel patterns were simulated by modelling either a body-centered cubic or simple cubic crystal with various lattice constants and calculating the diffraction pattern for a converging monochromatic light of wavelength 450 nm. Orthographic projections of the Kossel line corresponding to each pole were taken to obtain patterns corresponding to experiments. The refractive index of the BP was assumed to be 1.6.

## Supplementary information

Supplementary Information.
