# Supplementary material for: Orientation control of ideal blue phase photonic crystals
Source: Sci Rep. 2020 Jun 23;10:10148. doi: 10.1038/s41598-020-67083-6 (PMC7311397; doi:10.1038/s41598-020-67083-6)
Supplement: Supplementary file 1 — Supplementary Information. [file 41598_2020_67083_MOESM1_ESM.pdf]

Supplementary Information for:

Orientation control of ideal blue phase photonic crystals

Eva Oton[1]*, Hiroyuki Yoshida[2], Przemysław Morawiak[1], Olga Strzeżysz[1], Przemysław Kula[1], Masanori Ozaki[2], Wiktor Piecek[1]

[1]Institute of Applied Physics, Military University of Technology, gen. Witolda Urbanowicza 2, 00-908 Warsaw, Poland

[2]Division of Electrical, Electronic, and Information Engineering, Osaka University 2-1 Yamadaoka, Suita, Osaka 565-0871 Japan

e-mail: *eva.oton@wat.edu.pl*

1. Simulation of Kossel Patterns

The Kossel pattern technique is a crystallographic technique in which a monochromatic, divergent beam is illuminated on the target crystal [S1]. The technique was originally devised to analyze atomic crystals using X-rays, but can also be applied to visible light as long as the period of the crystal is comparable to the wavelength. Simulation of the Kossel diagrams was performed as outlined in ref. [S2], assuming body-centered-cubic and simple-cubic crystals of different lattice constants and orientations to match the image obtained in experiment. Below we show representative Kossel patterns of the BP samples and corresponding simulations, which confirmed the lattice plane orientations reported in the main manuscript (Fig S-1).

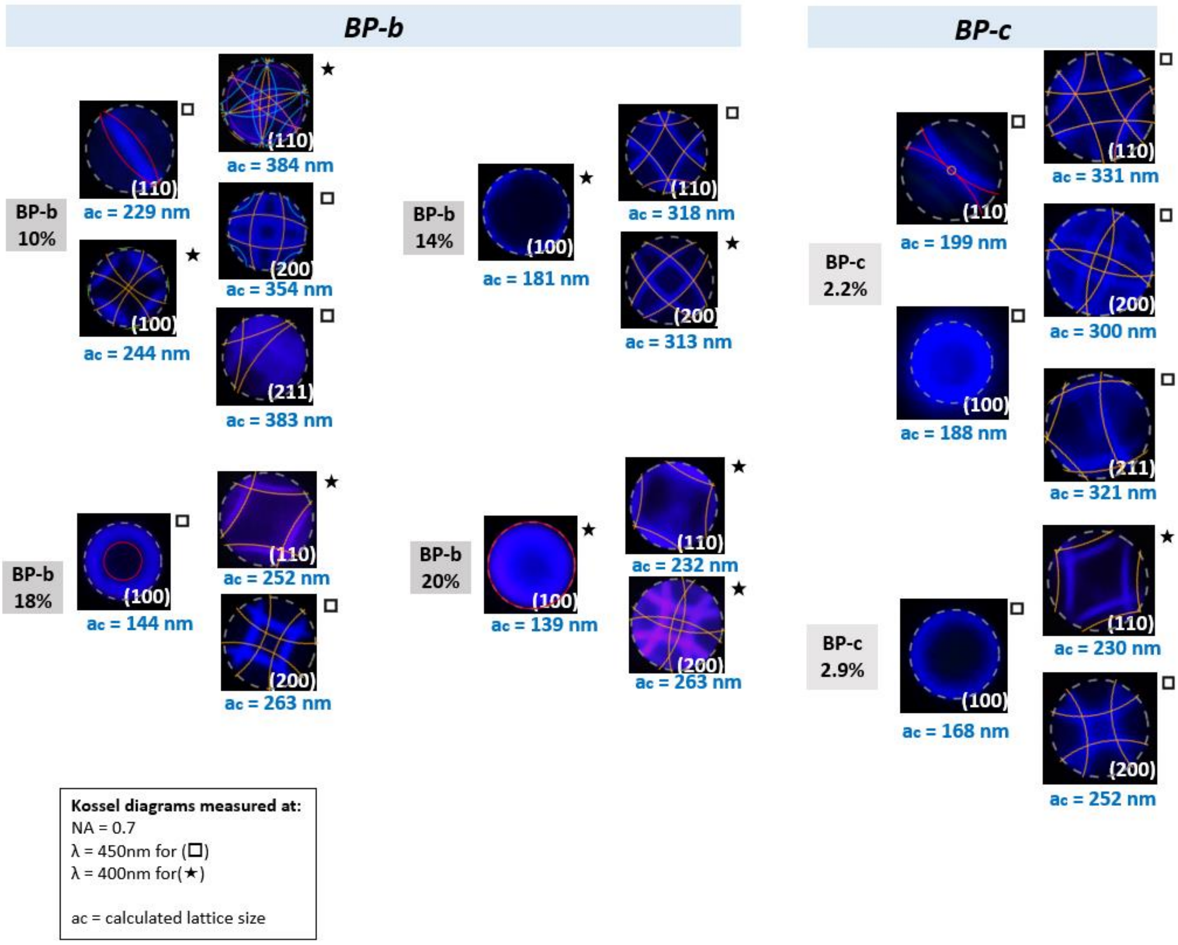

Fig S-1 – Kossel patterns and their corresponding simulation (thin lines) for some of the observed samples and their calculated lattice sizes.

2. Polyimide alignment layers tested for Blue Phases

Along with Nylon layers, a number of commercial polyimide layers with known strong anchoring conditions were prepared in glass substrates, rubbed in typical conditions for standard nematic mixtures and assembled into 5µm cells. As opposed to Nylon, none of the tested polyimides produced ideal BP monocrystals. In most cases, disorganized platelets or semi organized crystallites appeared (Fig S-2). To prevent very strong anchoring conditions, we tried several polyimide layers treated with very soft rubbing conditions, as well. The BPs obtained from those samples showed better homogeneity, some cells showed a few monocrystalline areas, although the layers still induced many defects and crystal misorientations. It is entirely possible that optimal rubbing parameters on certain polyimides might induce monocrystalline BPs, however in our case, all Nylon layers were significantly superior for any polyimide to be considered as an alternative.

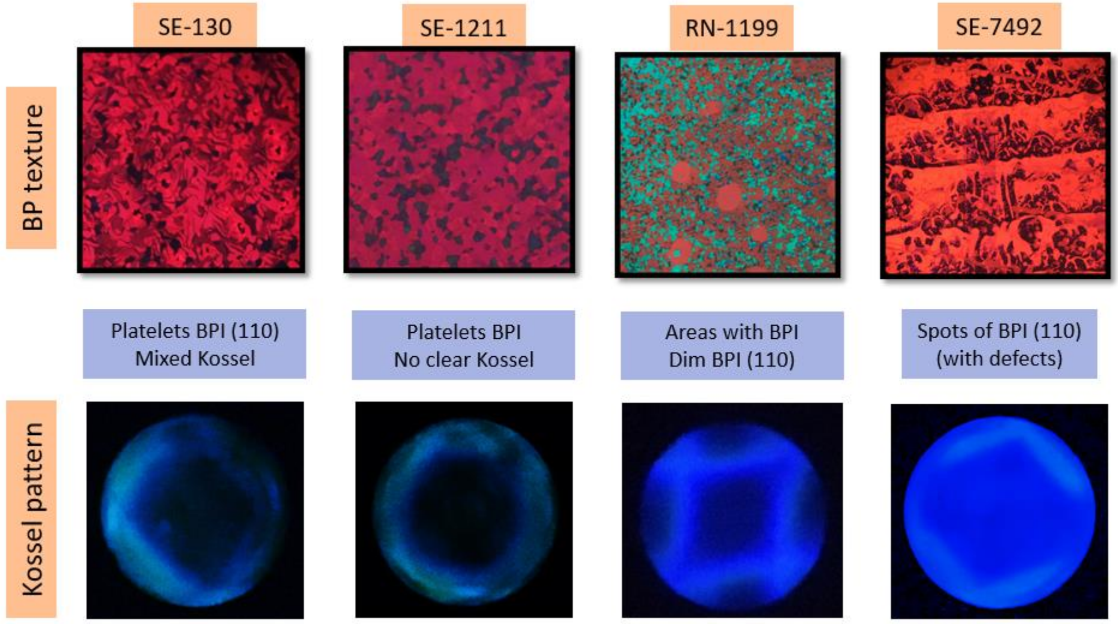

Fig S-2 – Polyimides that were tested as alignment layers for BPs.

[S1] Tixier, R., Wache, C., Kossel Patterns, J. Appl. Cryst. 3, 466–485 (1970).
[S2] Pieranski, P., Dubois-Violette, E., Rothen, F., Strzelecki, L., Geometry of Kossel lines in colloidal crystals, J. Physique 42, 53 (1981).
